# Bub1 kinase in the regulation of mitosis

**DOI:** 10.1080/19768354.2021.1884599

**Published:** 2021-02-17

**Authors:** Taekyung Kim, Anton Gartner

**Affiliations:** a Department of Biology Education, Pusan National University, Busan, Korea; b IBS Center for Genomic Integrity, Ulsan, Korea; c School of Life Sciences, Ulsan National Institute of Science and Technology

**Keywords:** Cell cycle, mitosis, kinetochores, spindle assembly checkpoint, Bub1

## Abstract

Accurate chromosome segregation is required for cell survival and organismal development. During mitosis, the spindle assembly checkpoint acts as a safeguard to maintain the high fidelity of mitotic chromosome segregation by monitoring the attachment of kinetochores to the mitotic spindle. Bub1 is a conserved kinase critical for the spindle assembly checkpoint. Bub1 also facilitates chromosome alignment and contributes to the regulation of mitotic duration. Here, focusing on the spindle assembly checkpoint and on chromosome alignment, we summarize the primary literature on Bub1, discussing its structure and functional domains, as well its regulation and roles in mitosis. In addition, we discuss recent evidence for roles of Bub1 beyond mitosis regulation in TGFβ signaling and telomere replication. Finally, we discuss the involvement of Bub1 in human diseases, especially in cancer, and the potential of using Bub1 as a drug target for therapeutic applications.

## Introduction

When cells divide, they need to equally segregate the duplicated genome to their daughter cells. Errors in chromosome segregation cause aneuploidy, a phenotype observed in most cancer cells and associated with birth defects (Santaguida and Amon 2015; Funk et al. 2016).

The kinetochore is a large multiprotein complex that assembles on chromosomes to mediate the interaction with mitotic spindle microtubules which facilitate chromosome segregation. At the kinetochore the ‘KMN’ network composed of the Knl1/Mis12 and the Ndc80 complexes mediates microtubule attachment. Besides being required for microtubule attachment, the ‘KMN’ network also coordinates orderly cell cycle progression by activating the spindle assembly checkpoint (SAC) when kinetochores are not attached to microtubules. The SAC halts cell cycle progression until all kinetochores are correctly attached. The target of the spindle assembly checkpoint is the Anaphase Promoting Complex/ Cyclosome (APC/C), a large E3 ubiquitin ligase that triggers the degradation of key substrates needed for chromatid separation and the execution of mitotic exit (for review see (Cheeseman and Desai 2008; Musacchio and Desai 2017)).

Bub1 (budding uninhibited by benzimidazole 1), is a conserved mitotic Serine/Threonine kinase first identified in budding yeast (Hoyt et al. 1991). In addition to the role in SAC signaling, Bub1 also promotes chromosome alignment and is involved in APC/C activation (Kim et al. 2015; Yang et al. 2015; Kim et al. 2017). Here, we first summarize basic mechanisms of the spindle assembly checkpoint. We then focus on Bub1, first describing its functional domains before elaborating how Bub1 regulates mitosis progression. Finally, we summarize recent evidence for roles of Bub1 outwith mitosis regulation.

## Spindle assembly checkpoint (SAC)

SAC components were initially identified via genetic screens conducted in budding yeast. Normally, when cells are treated with benzimidazole, a drug that disrupts spindle microtubules, cells arrest in mitosis. In contrast, mutants defective for SAC genes fail to arrest in mitosis. Bub1, Bub3, and the mitotic-arrest deficient (Mad) genes Mad1, Mad2, Mad3 (termed BubR1 in higher eukaryotes) were found In the initial screens; other SAC components such as Mps1 being identified later (Hoyt et al. 1991; Li and Murray 1991;Weiss and Winey 1996).

**Figure 1.**
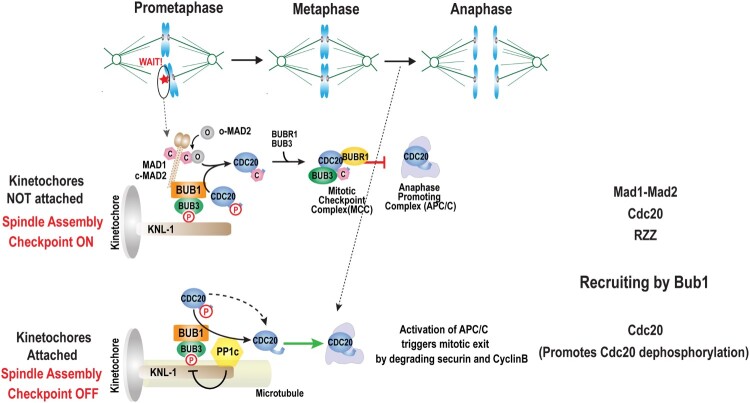
Overview of the spindle assembly checkpoint (SAC) pathway highlighting Bub1 mediated anaphase promoting complex (APC/C) regulation by Cdc20. Schematic of the spindle assembly checkpoint (Kim et al. 2017). When kinetochores are not attached to the microtubules, SAC components accumulate at the kinetochores and facilitate the formation of the mitotic checkpoint complex (MCC). The MCC inhibits the APC/C. Inactive APC/C fails to degrade cyclin B and Securin, preventing anaphase onset and subsequent mitotic exit. Bub1 contributes to the spindle assembly checkpoint by recruiting Mad1-Mad2, Cdc20. Once kinetochores are attached, APC/C activation is facilitated by the localized dephosphorylation of kinetochore bound Cdc20 mediated by PP1c.

The SAC was extensively reviewed (Lara-Gonzalez et al. 2012; Foley and Kapoor 2013; Musacchio 2015), and we therefore only provide a brief summary of the SAC (Figure 1). During mitosis, once all kinetochores are attached to the spindle microtubules, anaphase commences. During anaphase, chromatid separation is irreversibly triggered by Separase, a specialist protease that cleaves Cohesin rings encircling sister chromatids. Separase becomes activated when released from Securin, the critical target of APC/C dependent proteolytic degradation. APC/C activation and ensuing Securin degradation, Separase activation and Cohesin cleavage occur only when all kinetochores are attached to the spindle microtubules. In addition, inactivation of the master mitotic kinase Cdk1 is also dependent on APC/C dependent proteolytic degradation of Cyclin B, to ensure that anaphase onset and Cdk1 inactivation is tightly coupled.

APC/C inhibition is caused by the accumulation of SAC components at unattached kinetochores, a process that mediates the formation of the mitotic checkpoint complex (MCC) composed of Mad2, Bub3, BubR1, and Cdc20. A key step for SAC activation is the conformational switch of Mad2, from an open to a closed conformation, conferred by Mad1 residing at unattached kinetochores. Mad2 in its closed conformation binds to Cdc20, an essential co-activator of APC/C. This, together with the MCC complex, blocks the activation of APC/C (for reviews, Lara-Gonzalez et al. 2012; Foley and Kapoor 2013; Musacchio 2015). Once all kinetochores are attached to the spindle microtubules, all SAC components are removed from the kinetochore, leading the cessation of the checkpoint signal allowing for APC/C activation and anaphase progression (for reviews, Pesin and Orr-Weaver 2008; Pines 2011).

In addition to the core SAC components, other kinetochore proteins including the RZZ (Zw10/Rod/Zwilch) complex, the Aurora B-, Mps1- and Plk1 kinases, as well as the PP1 and PP2A phosphatases are involved in regulation of the SAC (Cheeseman and Desai 2008; Foley and Kapoor 2013). Focusing on Bub1, we will discuss how the Mps1 and Plk1 kinases, and the PP1 and PP2A phosphatases regulate Bub1, and how Bub1 may regulate RZZ at the kinetochore to facilitate checkpoint activation and normal chromosome segregation.

### Bub1 structure

**Figure 2.**
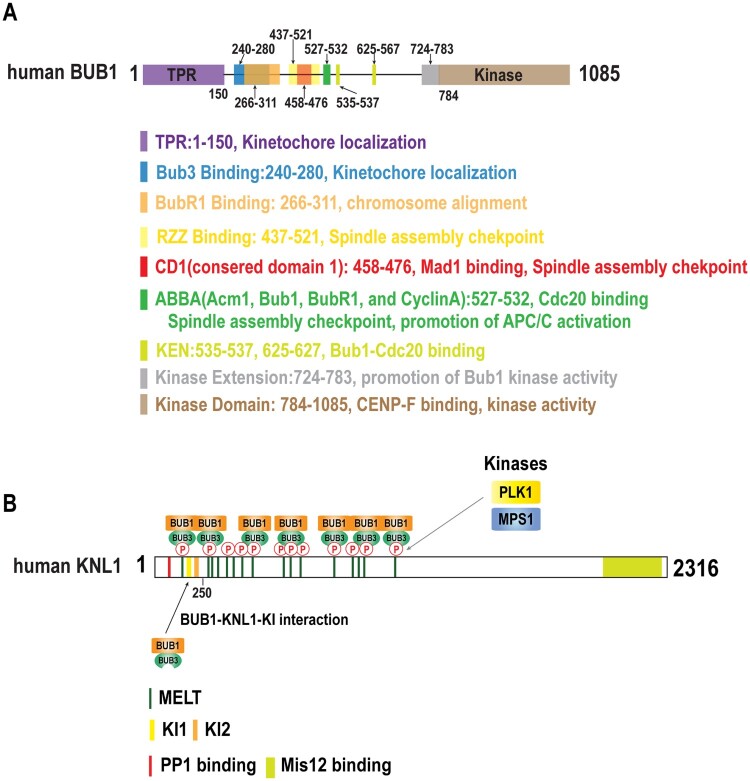
Domain structure of human Bub1 and KNL1. A. Bub1 domains. Conserved domains are highlighted by color coding, with the function of Bub1 domains being summarized below. B. KNL1 motifs and domains. Note that multiple ‘MELT’ phosphorylation motifs needed for Bub1/Bub3 recruitment reside in the N-terminal half of the protein.

X-ray crystal structures and extensive domain analyses revealed that Bub1 contains several conserved motifs essential for its functions in SAC activation and mitosis (Figure 2A). The N-terminal region of Bub1 is the most conserved region, and a crystal structure of a budding yeast N-terminal Bub1 fragment revealed a common domain that comprises a triple-tandem arrangement of tetratricopeptide repeat (TPR) motifs (Bolanos-Garcia et al. 2009), which also occur in a wide variety of other proteins including the p67-phox NADPH oxidase subunit, protein kinase R, and protein phosphatase 5 (Das et al. 1998). The N-terminal Bub1 TPR repeat containing domain was shown to interact with a 12-residue motif, termed KI (Lys-Ile)-motif residing in the N-terminal region of the KNL1 kinetochore protein (Figure 2) (Kiyomitsu et al. 2007; Kiyomitsu et al. 2011; Krenn et al. 2012; Krenn et al. 2013).

Following the N-terminal domain there is a conserved stretch of about 40 amino acid residues known as the ‘Bub3-binding motif’ originally described as ‘Gle20-binding sequence motif’ (Figure 2A) (Larsen et al. 2007; Primorac et al. 2013). Bub1 mutations that disrupt the Bub3-binding motif abolish Bub1 kinetochore localization and result in chromosome alignment defects. Conversely, mutations of Bub3 that disrupts the Bub1 and Bub3 interaction interface decreases Bub1 kinetochore localization (Taylor et al. 1998; Klebig et al. 2009). Therefore, Bub1 and Bub3 kinetochore localization are interdependent.

Bub1 also contains a central motif known as ‘ABBA motif’ (for Acm1, Bub1, BubR1, and cyclin A) or ‘Phe box’. Kinetochore recruitment of Cdc20, an event central to the SAC, depends on the interaction between the Bub1 ABBA motif and Cdc20 (Figure 2A) (Di Fiore et al. 2015; Diaz-Martinez et al. 2015). Interestingly, kinetochore localization of Cdc20 and its localized dephosphorylation (see below) are required for the subsequent promotion of APC/C activation (Kim et al. 2017). Thus, the transient localization of Cdc20 at the kinetochore is associated both with the inactivation and the activation of the APC/C, needed for cell cycle arrest and cell cycle progression respectively (also see below).

Mammalian Bub1 also contains two KEN boxes, degrons recognized by APC/C. Both KEN boxes are required for Bub1-Cdc20 binding (Figure 2A) (Tang et al. 2004; Qi and Yu 2007) The centrally located CD1 region (for conserved domain 1) was shown to recruit Mad1, an interaction critical for SAC activation (Figure 2A) (London and Biggins 2014; Zhang et al. 2017).

Finally, the C-terminal third of Bub1 contains a conserved Serine/Threonine kinase domain, preceded by a ‘N-terminal extension’. This latter domain uncovered by structural analysis promotes Bub1 kinase activity by stabilizing the active conformation of Bub1, analogous to CDK activation conferred by cyclin binding (Figure 2A) (Kang et al. 2008) The Bub1 kinase domain directly interacts with the CENP-F kinetochore protein. However, the function of the kinetochore-associated pool of CENP-F tethered by Bub1 is not clear (Ciossani et al. 2018).

## The function of Bub1 in the spindle assembly checkpoint and the promotion of APC/C activation

The function of Bub1 in the SAC is conserved from yeast to nematodes, insects and mammals (Hoyt et al. 1991; Basu et al. 1999; Meraldi and Sorger 2005; Essex et al. 2009). Bub1 activates the SAC by recruiting the Mad1-Mad2 complex to unattached kinetochores **(**Figures 1 and 2**)** (Klebig et al. 2009; London and Biggins 2014; Moyle et al. 2014). Bub1 derivates lacking the conserved domain I, fail to recruit Mad1 and Mad2 to the kinetochore (Klebig et al. 2009). In budding yeast and human cells, the central CD1 containing region of Bub1 can be phosphorylated by the conserved protein kinase Mps1. Phosphorylation of the corresponding Bub1 phosphorylation sites are required for Mad1 binding and SAC signaling (London and Biggins 2014; Ji et al. 2017; Zhang et al. 2017). In addition, Mps1 also contributes to SAC activation by phosphorylating the C-terminal region of Mad1, which promotes the interaction between Mad1 and Cdc20 and the assembly of the MCC (Faesen et al. 2017; Ji et al. 2017). The function of Bub1-CD1 to accurately localize Mad1 to the kinetochore, was revealed by experiments demonstrating that tethering of Mad1 or Mad2 to Bub1 bypasses the requirement of CD1 for SAC activation (Zhang et al. 2017). In contrast in *C. elegans*, the C-terminal BUB-1 kinase domain interacts with MAD-1 as demonstrated by the yeast two-hybrid method, and by point mutations disrupting the interaction interface of both proteins (Moyle et al. 2014). Thus, in *C. elegans,* the BUB-1 kinase domain is required for MAD-1 kinetochore recruitment.

In addition, the RZZ (Rod/Zw10/Zwilch) complex was suggested to contribute to the kinetochore binding of Mad1 in mammalian cells. Furthermore, human Bub1 was shown to recruit RZZ to the kinetochore, and tethering of Mad1 to kinetochores bypassed the requirement of RZZ for SAC signaling, suggesting that RZZ contributes to SAC activation by recruiting Mad1 to the kinetochore (Zhang et al. 2015; Zhang et al. 2019). In summary, Bub1 contributes to the SAC signaling by recruiting Mad1 to the kinetochore through direct and indirect interaction (Figures 1 and 2).

Bub1 leads to SAC activation at unattached kinetochores by recruiting Cdc20 to the kinetochore through the ABBA motif (Di Fiore et al. 2015; Diaz-Martinez et al. 2015; Kim et al. 2017). Mutation of the ABBA motif abolishes Cdc20 kinetochore localization and abrogates SAC activity without affecting Mad1/Mad2 recruitment (Di Fiore et al. 2015; Diaz-Martinez et al. 2015; Kim et al. 2017). The ABBA motif of Bub1 is close to the CD1 region of Bub1 that recruits Mad1 (Figure 2A). It is therefore likely that the kinetochore recruitment Cdc20 through the ABBA motif of Bub1 brings Cdc20 close to Mad1/Mad2, thereby promoting the formation of the MCC that inhibits the APC/C. In addition, Bub1 contributes to SAC activation by directly phosphorylating Cdc20 recruited to the kinetochore, and indirectly by facilitating Cdc20 phosphorylation by recruiting the Plk1 Polo kinase (Tang et al. 2004; Jia et al. 2016).

Unexpectedly (Figure 1, lower section), mutation of the BUB-1 ABBA motif in *C. elegans* not only abolished SAC signaling but also delayed anaphase onset (Kim et al. 2017). A function of Bub1 in promoting anaphase onset was also reported in budding yeast (Yang et al. 2015). Cdc20 contains conserved Cdk1 phosphorylation sites at its N-terminus, and a non-phosphorylatable *C. elegans* derivate of CDC-20 accelerated mitosis via precocious APC/C activation (Kim et al. 2017). In line with prior *in vitro* studies (Kramer et al. 2000; Yudkovsky et al. 2000; Labit et al. 2012; Hein and Nilsson 2016), this result suggests that Cdc20 phosphorylation by Cdk1 inhibits binding to and activation of APC/C. The non-phosphorylatable derivate of CDC-20 bypassed the need for CDC-20 kinetochore localization for promoting APC/C activation, suggesting that CDC-20 dephosphorylation at the kinetochore is required for the promotion of APC/C. This dephosphorylation is mediated by the KNL-1-associated PP1c phosphatase, supported by experimental evidence showing that perturbing PP1c or CDC-20 kinetochore localization equally delays anaphase onset (Kim et al. 2017). The half-life of Cdc20 at kinetochores is very short indicative of rapid turnover rates (Howell et al. 2004; Kallio et al. 2002; Kim et al. 2017). The rapid flux of Cdc20 through the kinetochore might be part of two opposing mechanisms. Cdc20 is either incorporated into the MCC to inhibit APC/C in response to unattached kinetochores, while promoting Cdc20 dephosphorylation at attached kinetochores may promote APC/C activation and cell cycle progression. As SAC signaling is activated at unattached kinetochores, microtubule attachment is likely to control the fates of Cdc20. (Figure 1) Although the precise mechanism is not clear, this may involve the dephosphorylation of Cdc20 by phosphatase PP1, the activity of which at the kinetochore is regulated by microtubule attachment (Liu et al. 2010; Lesage et al. 2011; London et al. 2012).

A key question of SAC activation, relates to the problem of how a single unattached kinetochore leads to the inhibition of all APC/C. The rapid flux of Cdc20 through the kinetochore might be part of a signal amplification mechanism, where abundant Cdc20 protein is cycled through the kinetochore to allow for the stoichiometric inhibition of APC/C in response to unattached kinetochores. A mechanism which is countered by dephosphorylated Cdc20 generated at spindle microtubule-attached kinetochores.

## Kinetochore localization mechanism of Bub1

To act as a scaffold Bub1 needs to be recruited to the kinetochore for checkpoint signaling and chromosome alignment. Bub1 is amongst the first checkpoint components to dock at the nascent kinetochore, and kinetochore binding is stable based on fluorescence photobleaching recovery (FRAP) experiments (Howell et al. 2004; Rischitor et al. 2007). The Bub1/Bub3 complex is recruited to the kinetochore by the core outer kinetochore component KNL1 via multiple ‘MELT (Met-Glu-Leu-Thr)’ motifs which upon being phosphorylated by Mps1 and Plk1 mediate Bub1/Bub3 recruitment (London et al. 2012; Shepperd et al. 2012; Yamagishi et al. 2012). Mechanistic details of this interaction were uncovered by a co-crystal structure of the ScBub1^258-359^/Bub3 complex in conjunction with a phosphorylated MELT peptide (p-MELT) (Primorac et al. 2013): Conserved Bub3 residues directly interact with the phosphorylated MELT motif and Bub1 contributes to this interaction; the binding affinity between Bub3 and p-MELT being decreased ∼10-fold in the absence of Bub1. Based on these results, the current model posits that in early mitosis, Mps1 and Plk1 kinases recruit Bub1/Bub3 complex by phosphorylating tandem ‘MELT’ motifs residing in the KNL1 outer kinetochore protein.

Once the Bub1/Bub3 complex interacts with KNL1, the SAC can be initiated. At the same time Bub1/Bub3 recruitment facilitates faithful chromosome segregation by mechanisms that are not entirely clear (Figure 2B).

The phospho-dependent recruitment of Bub1/Bub3 complex to kinetochore bound KNL1 is antagonized by the PP1c phosphatase which is equally recruited to KNL1 via a separate interaction domain. Substitutions of conserved KNL-1 residues within the *C. elegans* PP1c interaction domain perturb PP1c kinetochore recruitment and increase kinetochore localization of the BUB-1/BUB-3 complex*,* indicating that PP1c activity restricts the extent of MELT motif phosphorylation (Kim et al. 2017). Although mechanisms are not clear, PP1c and PP2A-B56 phosphatase activity might be equally increased at kinetochores to dephosphorylate KNL1. Thus, the dephosphorylation of KNL1 might be part of a mechanism that silences the SAC once kinetochores are attached to the spindle microtubules. The phospho-specific interaction between Bub1/Bub3 complex and KNL1 appears to be supported by a secondary interaction surface provided by the N-terminal TPR region of Bub1 (Kiyomitsu et al. 2007; Kiyomitsu et al. 2011; Krenn et al. 2012; Krenn et al. 2013) (Figure 2A). While this interaction was confirmed by interaction studies and structural evidence, abrogating this secondary interaction does not perturb Bub1 kinetochore recruitment (Krenn et al. 2013). Given that the KNL1-KI motif that mediates Bub1 binding is poorly conserved, the interaction between the Bub1-TPR motif and the KNL1 KI-motif may only function in higher eukaryotes, and may only be used for the fine-tuning.

## The function of Bub1 in chromosome segregation

In addition to its function in recruiting checkpoint components Mad1 and Cdc20, Bub1 also recruits a number of other kinetochore proteins including Mad3/BubR1, the centromere protein F (Cenp-F), and RZZ (Figure 2A) (Johnson et al. 2004; Kiyomitsu et al. 2007; Klebig et al. 2009). siRNA-mediated depletion of BUB1 was shown to result in chromosome alignment defects and lagging chromosomes in human cells and other systems including yeasts and *C. elegans* (Bernard et al. 1998; Gillett et al. 2004; Meraldi and Sorger 2005; Kim et al. 2015).

Mad3/BubR1 is a core SAC protein which localizes to the kinetochore through the interaction with Bub1. Bub1 directly interacts with BubR1 *in vitro*, the interaction requiring the central amino acids Bub1 266–311 region (Overlack et al. 2014; Zhang et al. 2015). Kinetochore localization of BubR1 is not essential for a functional SAC signal (Essex et al. 2009; Kulukian et al. 2009; Malureanu et al. 2009), but it is required for the chromosome alignment (Lampson and Kapoor 2005; Matsumura et al. 2007; Suijkerbuijk, Vleugel, et al. 2012). During mitosis, counteracting kinases and phosphatases work together to accurately mold kinetochore-microtubule interactions and to progressively stabilize them during mitosis progression. Phosphatase PP2A-B56 antagonizes Aurora B activity at the kinetochore to regulate kinetochore-microtubule interactions (Foley et al. 2011; Saurin 2018). BubR1 contains a motif referred to as KARD (Kinetochore Attachment Regulatory Domain), which interacts with protein phosphatase2A-B56 α (PP2A-B56α), and mutation of the BubR1 KARD domain showed an increased rate chromosome misalignment (Suijkerbuijk, Vleugel, et al. 2012). In addition, a BubR1 mutant, BubR1 (Δ432-484) which does not affect the KARD domain is impaired for kinetochore localization, and the corresponding cells showed defects in promoting the formation of stable kinetochore-microtubule attachments (Overlack et al. 2014).

Bub1 also recruits CENP-F to the kinetochore (Johnson et al. 2004; Cheeseman et al. 2005; Boyarchuk et al. 2007; Klebig et al. 2009; Ciossani et al. 2018). Direct interaction between the Bub1 kinase domain and CENP-F was shown *in vitro* (Ciossani et al. 2018)*,* and expression of the Bub1 lacking its kinase domain abolished kinetochore localization of CENP-F in human HAP1 cells (Raaijmakers et al. 2018). Although depletion CENP-F in human cells only leads to very mild mitotic defects (Raaijmakers et al. 2018), the Bub1 lacking kinase domain results in chromosome alignment defects (Klebig et al. 2009; Raaijmakers et al. 2018). These results indicate that the Bub1 C-terminal kinase domain may recruit other downstream targets. Interestingly, in *C. elegans*, BUB-1-dependent CENP-F^HCP-1/HCP-2^ kinetochore-localization promotes the formation of the central spindle during anaphase (Maton et al. 2015).

Lastly, Bub1 contributes to the recruitment of Rod-Zwilch-ZW10 (RZZ) in human cells. RZZ mediates the recruitment of dynein-dynactin to kinetochores which may be required for chromosome alignment (Griffis et al. 2007). However, Bub1 Δ437-521 cells which show a ∼70% reduction of RZZ kinetochore localization, are nevertheless able to fully rescue chromosome alignment (Zhang et al. 2015; Raaijmakers et al. 2018), indicating that Bub1 dependent RZZ recruitment may not be a significant factor for regulating chromosome alignment.

## Bub1 kinase function

The kinase activity of Bub1 is highly conserved. Nevertheless, only two direct substrates of the Bub1 kinase have been identified. Bub1 was shown to directly phosphorylate Cdc20 *in vitro*, and this phosphorylation may directly contribute to the SAC signaling (Tang et al. 2004). Importantly, Bub1 phosphorylates histone H2A at a conserved residue corresponding to threonine 121 in yeast to create a binding site for MEI-S32/Shugoshin (Sgo) proteins, which in turn recruit the chromosomal passenger complex (CPC) composed of Aurora B, inner centromere protein (INCENP), Survivin and Borealin to the centromere (Kitajima et al. 2005; Kawashima et al. 2010; Liu et al. 2013; Liu et al. 2015). The CPC complex includes the Aurora B kinase, which plays a role in microtubule attachment and SAC signaling (Krenn and Musacchio 2015). Also, in mammals, Sgo1 recruited by Bub1 forms a complex with protein phosphatase 2A (PP2A), which in turn protects centromeric chromatid cohesion at up to the metaphase-anaphase transition (Tang et al. 2006; Eshleman and Morgan 2014). Although Bub1 kinase activity contributes to faithful chromosome segregation, Bub1 inactivation only leads to mild defects in human cells. Treating HeLa and RPE1 cells with Bub1 kinase inhibitors leads to a ∼80% reduction of Sgo1 and Sgo2, and the CPC subunits Aurora B and Borealin at centromeres; INCENP being reduced by half and Aurora B being redistributed to the chromosome arms (Baron et al. 2016).

In addition, as the microtubule dynamics regulator Mitotic Centromere-Associated Kinesin (MCAK) is regulated by Aurora B, inhibition of Bub1 kinase activity reduced MCAK levels by ∼50% at the inner centromere (Baron et al. 2016). However, Bub1 inhibition did not show a significant impact on mitotic progression and SAC signaling (Baron et al. 2016). Given that a second pathway involving the Haspin kinase also contributes to the recruitment of the CPC to inner centromeres (Dai et al. 2006; Wang et al. 2011), inhibiting Bub1 kinase activity alone may not have a drastic effect on mitotic progression.

Interestingly, it was recently suggested that Bub1 might also have roles independent of mitosis. It was shown that Bub1 promotes TGF-β signaling in mouse tumor tissues and that this is dependent on Bub1 kinase activity (Nyati et al. 2015). In addition, it was suggested that the Bub1-Bub3 complex is targeted to telomeres by binding to telomere-binding protein TRF2 during S phase. Bub1 phosphorylates TRF1, which in turn promotes the recruitment of the Blooms DNA helicase which is need to overcome replication stress (Li et al. 2018). It will be interesting to explore Bub1 substrates beyond mitosis regulation.

### Bub1 in cancer relevance

Aneuploidy, a common feature in most cancer cells, is caused by an increased rate of chromosome mis-segregation tightly associated with mitotic errors (Holland and Cleveland 2012; Santaguida and Amon 2015; Funk et al. 2016). Bub1 mutations, including deletions and point mutations, as well as differential Bub1 gene expression have been implicated in carcinogenesis (Cahill et al. 1998; Ohshima et al. 2000; Hernando et al. 2001; Ru et al. 2002; Shichiri et al. 2002; Klebig et al. 2009; Wang et al. 2015). Bub1 overexpression in mice resulted in aneuploidy and tumor formation likely mediated through Aurora B hyperactivation (Ricke et al. 2011). In addition, overexpression of BUB1 and the MAD2 related protein MAD2L1 is associated with aggressive tumors in breast cancer (Wang et al. 2015), suggesting that dysregulation of SAC signaling may be involved in chromosome instability and aneuploidy. However, given the multiple roles of Bub1, it is not clear which Bub1 function is critical for tumorigenesis.

Although treating cells with the Bub1 kinase inhibitor BAY-320 or BAY-524 alone showed only minor effects in mitotic progression or SAC function, combined treatment with low doses of microtubule poison Paclitaxel impaired chromosome segregation and cell proliferation (Baron et al. 2016). These synergistic effects are particularly drastic in highly aneuploid HeLa cells, suggesting a potential use of Bub1 inhibitors for specifically killing aneuploid cancer cells.

## Final remarks

Since the initial discovery of the Bub1 and spindle assembly checkpoint genes in the early 1990s, Bub1 has been extensively studied by structural, biochemical, and cell-biological approaches. Bub1 contains several conserved domains, and these domains contribute to SAC signaling and to chromosome alignment by recruiting Mad1-Mad2, Cdc20, RZZ, CENP-F, and BubR1/Mad3, and by phosphorylating histone H2A and Cdc20. Interestingly, Bub1 is not only required for mitosis but also plays important roles outwith mitosis, promoting TGF-β signaling and telomeric genome integrity by BLM helicase recruitment. If and how Bub1 contributes to further processes not directly related to mitosis will be interesting avenues of further investigation. Finally, a better understanding the mechanism of Bub kinase activation and the identification of further substrates during SAC activation and normal cell cycle progression will facilitate the development of new approaches for specifically killing cancer cells.
